# Circulating Maresin-1 and cartilage remodeling biomarkers in rheumatoid arthritis and osteoarthritis

**DOI:** 10.1038/s41598-026-42927-9

**Published:** 2026-03-18

**Authors:** Omer Esmez, Gulnihal Deniz, Zubeyde Ercan, Secil Yılmaz, Meryem Sedef Dogru, Ahmet Karatas

**Affiliations:** 1 Department of Orthopedics, Elazığ Fethi Sekin City Hospital, 23100 Elazığ, Turkey; 2 Department of Physiotherapy and Rehabilitation, Faculty of Health Sciences, Erzurum Technical University, 25055 Elazığ, Turkey; 3 Department of Physiotherapy and Rehabilitation, Faculty of Health Sciences, Fırat University, Elazığ, Turkey; 4 Department of Physiology, Faculty of Medicine, Fırat University, Elazığ, Türkiye Turkey; 5 Division of Rheumatology, Department of Internal Medicine, Faculty of Medicine, Fırat University, 23119 Elazığ, Turkey

**Keywords:** Maresin-1, Rheumatoid Arthritis, Osteoarthritis, Cartilage Oligomeric Matrix Protein, WISP-1, Biomarker, Biomarkers, Diseases, Immunology, Medical research, Rheumatology

## Abstract

Rheumatoid arthritis (RA) and osteoarthritis (OA) are prevalent chronic joint diseases characterized by persistent inflammation, progressive cartilage degeneration, and impaired tissue repair mechanisms. While inflammatory pathways have been extensively studied, increasing attention has been directed toward the failure of inflammation resolution as a key contributor to chronic joint pathology. Specialized pro-resolving mediators (SPMs), including Maresin-1 (MaR1), play an active role in terminating inflammation and promoting tissue homeostasis. However, the relationship between MaR1 and cartilage-specific biomarkers such as Cartilage Oligomeric Matrix Protein (COMP) and Wnt1-inducible signaling pathway protein-1 (WISP-1) has not been sufficiently elucidated. This study investigated circulating MaR1 levels in RA and OA and examined their associations with serum COMP and WISP-1 as well as clinical inflammatory parameters. This cross-sectional, biomarker-based study included a total of 150 participants, comprising 50 patients with RA, 50 patients with OA, and 50 sex-matched healthy controls. Serum levels of MaR1, COMP, and WISP-1 were measured using enzyme-linked immunosorbent assay (ELISA). Demographic characteristics and inflammatory parameters, including erythrocyte sedimentation rate (ESR), C-reactive protein (CRP), and rheumatoid factor (RF), were recorded. Intergroup comparisons were performed using one-way analysis of variance (ANOVA) followed by appropriate post-hoc tests. Serum MaR1 levels were significantly reduced in both RA (44.1 ± 2.2 ng/L) and OA (42.2 ± 3.2 ng/L) compared with controls (78.5 ± 7.5 ng/L; *p* < 0.001). COMP levels were significantly lower in the RA group than in the OA and control groups (*p* < 0.05 and *p* < 0.001, respectively). Similarly, WISP-1 concentrations were decreased in both RA and OA compared with healthy controls (*p* < 0.05). The marked reduction in MaR1 levels in RA and OA is consistent with altered inflammation-resolution biology. Concurrent decreases in COMP and WISP-1 may reflect changes in cartilage remodeling dynamics. Overall, MaR1 appears to be a candidate biomarker associated with inflammation-resolution pathways in arthritic diseases.

**Supplementary Information:**

The online version contains supplementary material available at 10.1038/s41598-026-42927-9.

## Introduction

RA is a chronic, systemic autoimmune disorder associated with high morbidity and disability worldwide^1^. The inability of inflammation to resolve spontaneously results in persistent synovitis, progressive joint destruction, and lifelong disease activity. Understanding the mechanisms that regulate immune responses and promote inflammation resolution is therefore critical for identifying novel therapeutic targets and improving treatment strategies.

OA, traditionally considered a degenerative joint disease, is now recognized to involve chronic low-grade inflammation triggered by micro- or macro-traumatic joint injuries^2^. These molecular disturbances disrupt cartilage homeostasis, leading to matrix degradation, structural deterioration, and loss of function. Although anti-inflammatory drugs such as corticosteroids and NSAIDs are widely used, their long-term use is limited by adverse effects, highlighting the need for safer, resolution-oriented treatment modalities.

MaR1, a specialized pro-resolving lipid mediator derived from docosahexaenoic acid (DHA), was first identified in human macrophages^3^. MaR1 exhibits potent anti-inflammatory and tissue-protective effects by suppressing neutrophil migration, reducing cytokine release from Th1, Th17, and CD8⁺ T cells, and promoting macrophage polarization toward an M2 phenotype^4,5^. Several studies have demonstrated that reduced MaR1 concentrations in synovial fluid correlate with disease activity in RA, while MaR1 supplementation suppresses synovial inflammation and supports cartilage integrity in arthritis models^6,7^.

In OA, MaR1 has been shown to increase intra-articular concentrations and alleviate cartilage damage in animal models subjected to treadmill-induced joint stress^8^. Experimental evidence has demonstrated that MaR1 administration can enhance type II collagen synthesis and suppress MMP-13 expression in synovial tissues, indicating its involvement in pro-resolving and remodeling pathways^9^. Collectively, these findings highlight MaR1 as a bioactive mediator capable of both limiting inflammation and promoting tissue regeneration.

Among biomarkers reflecting cartilage integrity, COMP and WISP-1 (also known as CCN4) hold particular importance. COMP is a structural glycoprotein reflecting cartilage matrix turnover and has prognostic value in both OA and RA^10–12^. WISP-1, a member of the CCN protein family, regulates extracellular matrix remodeling, chondrocyte survival, and inflammatory signaling^13^. Increased WISP-1 expression has been associated with synovial inflammation and cartilage degeneration in both OA and RA^14–16^.

Given these findings, investigating the association between MaR1, COMP, and WISP-1 may provide insight into the interplay between defective inflammation resolution and cartilage degeneration. Therefore, this study aimed to examine whether circulating MaR1 levels are associated with alterations in cartilage remodeling biomarkers in RA and OA.

## Materials and methods

### Study design and patient selection

This cross-sectional study included a total of 150 participants, divided equally into three groups: 50 patients with RA, 50 patients with OA, and 50 healthy controls. Participants were recruited from the Orthopedics and Traumatology Department of Elazığ Fethi Sekin City Hospital and the Rheumatology Department of Fırat University Hospital.

The study protocol was approved by the Fırat University Non-Interventional Research Ethics Committee (Session No: 2025/11–18, Date: August 21, 2025; Chair: Prof. Dr. Mustafa Kaplan). Written informed consent was obtained from all participants prior to enrollment, and all study procedures were conducted in accordance with the principles of the Declaration of Helsinki.

Patients in the OA group were aged between 40 and 70 years and had radiographically confirmed Kellgren–Lawrence grade 4 knee osteoarthritis. The RA group consisted of individuals diagnosed according to the 2010 American College of Rheumatology (ACR) and/or ACR–EULAR classification criteria. The control group included healthy volunteers aged ≥ 50 years with no clinical or radiographic evidence of osteoarthritis. The control group was frequency-matched to the patient groups by sex. Although age distributions differed between groups, potential confounding due to age was addressed using age-adjusted statistical analyses.

Exclusion criteria were malignancy, diabetes mellitus, hypertension, hyperlipidemia, hepatic, renal, or cardiac disease, other systemic autoimmune disorders, and recent bacterial or viral infections.

Information regarding ongoing pharmacological treatment was obtained from medical records. Patients with RA were receiving standard-of-care therapy, including conventional disease-modifying antirheumatic drugs (DMARDs) and/or biologic agents. None of the participants were receiving high-dose corticosteroids or experiencing acute disease flares at the time of blood sampling. OA patients were treated with intermittent non-steroidal anti-inflammatory drugs as needed. Blood samples were collected during routine outpatient follow-up visits under clinically stable conditions.

### Participants and power analysis

Sample size estimation was performed using G*Power software (version 3.1.16). Assuming a medium effect size (Cohen’s *f* = 0.25), a significance level of α = 0.05, and statistical power (1–β) = 0.80, a minimum total sample size of 150 participants (50 per group) was calculated to be sufficient to detect significant intergroup differences using one-way analysis of variance (ANOVA) (df_1_ = 2, df_2_ = 147; critical *F* ≈ 3.06).

### Outcome measures

Demographic characteristics, including age, sex, height, weight, body mass index (BMI), and disease duration, were recorded for all participants. Laboratory parameters assessed included ESR, CRP, and RF. Disease activity in patients with RA was evaluated using the DAS28.

Fasting venous blood samples (5 mL) were obtained from all participants between 08:00 and 10:00 a.m. following an overnight fast. Blood samples were centrifuged at 4000 rpm for 5 min at 4 °C, and the separated sera were stored at − 80 °C until further analysis.

Serum levels of MaR1, COMP, and WISP-1 were measured using commercially available enzyme-linked immunosorbent assay (ELISA) kits (Cloud-Clone Corp., Wuhan, China) in accordance with the manufacturer’s instructions. All samples were analyzed in duplicate, and intra-assay and inter-assay coefficients of variation were below 10%.

### Statistical analysis

Statistical analyses were conducted using SPSS software version 22.0 (IBM Corp., Armonk, NY, USA). The normality of continuous variables was assessed using the Shapiro–Wilk test. Normally distributed variables were analyzed using one-way analysis of variance (ANOVA), followed by Bonferroni-adjusted post-hoc comparisons where appropriate. Non-normally distributed variables were evaluated using the Kruskal–Wallis test with Dunn’s post-hoc correction. For ANOVA analyses, effect sizes were quantified using eta-squared (η^2^), calculated from the corresponding F-statistics to estimate the proportion of total variance attributable to between-group differences. η^2^ values of 0.01, 0.06, and ≥ 0.14 were interpreted as small, medium, and large effects, respectively. In addition to p-values, 95% confidence intervals (CI) were calculated for mean differences between each patient group and controls to provide estimates of precision. Degrees of freedom are reported in the Results section. Continuous variables are presented as mean ± standard deviation (SD). A two-tailed p-value < 0.05 was considered statistically significant.

To account for potential confounding due to age differences between study groups, an analysis of covariance (ANCOVA) was additionally performed with age included as a covariate in intergroup comparisons of MaR1, COMP, and WISP-1 levels.

In addition to ANCOVA analyses, multivariable linear regression models were constructed further to evaluate potential confounding effects of demographic and anthropometric variables. Serum MaR1, COMP, and WISP-1 levels were entered as dependent variables in separate models. Independent variables included age, sex, BMI, and disease group (dummy-coded). Standardized beta coefficients (β), 95% confidence intervals (CI), and p-values were calculated. Model fit was assessed using adjusted R^2^ values. Multicollinearity was evaluated using variance inflation factors (VIF), with values < 2.0 considered acceptable. Given the observed age differences between groups, age-adjusted analyses were considered primary for interpretation of intergroup biomarker comparisons.

The demographic and clinical characteristics of the study population, including age, sex distribution, body mass index, disease duration, and inflammatory markers (ESR, CRP, and RF), are summarized in Table 1.

Correlation analyses were performed separately in the RA and OA cohorts to assess relationships between serum MaR1, COMP, and WISP-1 levels and clinical parameters (ESR, CRP, DAS28 [RA only], and disease duration) using Pearson’s correlation coefficient (r); a two-tailed p-value < 0.05 was considered statistically significant.

## Results

A total of 150 individuals were evaluated in the study. The mean age of participants was 59.0 ± 9.5 years in the RA group, 61.5 ± 9.5 years in the OA group, and 51.2 ± 9.5 years in the control group. BMI (kg/m^2^), disease duration (years), and laboratory parameters including ESR (mm/h), RF (IU/mL), and CRP (mg/L) were recorded for all participants.The demographic and clinical characteristics of the study population are presented in Table 1.

**Table 1:** Demographic and biochemical characteristics of the study groups (Mean ± SD).

Parameters	Control (*n* = 50)	OA (*n* = 50)	RA (*n* = 50)
Age (years)	51.2 ± 9.5	61.5 ± 9.5	59.0 ± 9.5
Female / Male (%)	34/16 (68/32)	33/17 (66/34)	37/13 (74/26)
Mean Age (Female)	49.5 ± 9.3	59.9 ± 10.4	59.0 ± 9.1
Mean Age (Male)	51.3 ± 10.4	64.6 ± 6.7	59.1 ± 11.0
BMI (kg/m^2^)	24.8 ± 2.3	27.5 ± 3.1	26.9 ± 2.8
Disease duration (years)	—	8.2 ± 2.5	10.1 ± 3.6
ESR (mm/h)	12.4 ± 4.8	28.5 ± 9.7	42.6 ± 14.3
RF (IU/mL)	8.6 ± 3.1	21.8 ± 7.4	68.9 ± 26.5
CRP (mg/L)	3.1 ± 1.0	8.5 ± 3.5	18.7 ± 6.2

Data are expressed as mean ± standard deviation (SD).

*OA* osteoarthritis, *RA* rheumatoid arthritis, *ESR* erythrocyte sedimentation rate, *RF* rheumatoid factor, *CRP* C-Reactive Protein.

**Fig. 1:**
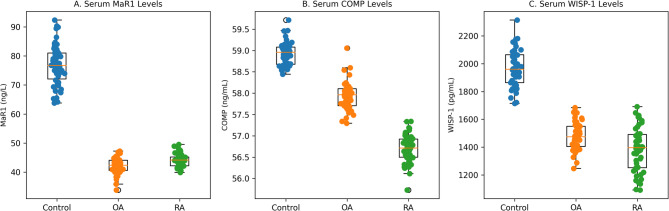
Distribution of serum MaR1, COMP, and WISP-1 levels across study groups. Box-and-whisker plots illustrate median values, interquartile ranges (IQRs), and the full data distribution, with individual data points overlaid. Between-group comparisons were performed using one-way analysis of variance (ANOVA) followed by Bonferroni post hoc testing. Exact p-values and effect sizes (η^2^) are reported in the Results section.

This study compared serum MaR1, COMP, and WISP-1 levels in patients with RA and OA and in healthy controls (Fig. 1).

Table 2 summarizes the intergroup comparisons of these biomarkers. MaR1 levels were significantly lower in both RA (44.1 ± 2.2 ng/L) and OA (42.2 ± 3.2 ng/L) groups than in controls (78.5 ± 7.5 ng/L; *p* < 0.001), consistent with reduced pro-resolving mediator levels in arthritic conditions. COMP concentrations did not differ significantly between OA (57.9 ± 0.3 ng/mL) and controls (58.9 ± 0.3 ng/mL). However, a modest directional reduction was observed in the OA group relative to controls, suggesting a limited but potentially biologically relevant variation. In contrast, RA patients exhibited significantly reduced COMP levels (56.7 ± 0.3 ng/mL) compared with both OA (*p* < 0.05) and control groups (*p* < 0.001). This pattern suggests that cartilage-related biomarker profiles may differ between RA and OA. WISP-1 levels were also significantly lower in RA (1392.0 ± 145.3 pg/mL) and OA (1461.7 ± 101.4 pg/mL) compared with controls (1959.4 ± 169.5 pg/mL; *p* < 0.05), reflecting altered extracellular-matrix remodeling. One-way ANOVA revealed significant between-group differences in serum MaR1 levels (F(2,147) = 19.654, *p* < 0.001; η^2^ = 0.211), indicating a large effect size. Compared with controls, mean MaR1 concentrations were reduced by − 34.4 ng/L in the RA group (95% CI: −36.6 to − 32.2) and − 36.3 ng/L in the OA group (95% CI: −38.6 to − 34.0). Significant differences were also observed for COMP (F(2,147) = 9.601, *p* < 0.001; η^2^ = 0.116), corresponding to a moderate-to-large effect size; the RA group demonstrated a mean reduction of − 2.2 ng/mL relative to controls (95% CI: −2.32 to − 2.08). Similarly, WISP-1 levels differed significantly among groups (F(2,147) = 4.802, *p* = 0.013; η^2^ = 0.061), reflecting a moderate effect size. Relative to controls, mean WISP-1 concentrations were lower by − 567.3 pg/mL in RA (95% CI: −629.9 to − 504.7) and − 497.7 pg/mL in OA (95% CI: −553.1 to − 442.3) (Table 2).

When age was included as a covariate in ANCOVA analysis, the between-group differences in MaR1 levels remained statistically significant (*p* < 0.001). Similarly, the differences observed for COMP (*p* < 0.01) and WISP-1 (*p* < 0.05) persisted after age adjustment, indicating that the observed biomarker alterations were not solely attributable to age-related effects.

Multivariable linear regression analyses adjusting for age, sex, and BMI demonstrated that disease group remained an independent predictor of MaR1 levels (β = − 0.58, 95% CI: − 0.71 to − 0.44, *p* < 0.001). The model explained 46% of the variance in MaR1 concentrations (adjusted R^2^ = 0.46). Neither sex (*p* = 0.39) nor BMI (*p* = 0.28) showed statistically significant independent associations. Variance inflation factors were below 2.0, indicating no evidence of multicollinearity. Similar results were observed for COMP and WISP-1, with disease status remaining the primary determinant of biomarker variability after multivariable adjustment.

Overall, serum MaR1 (a key lipid mediator involved in inflammation resolution) was markedly decreased in RA and OA, while COMP and WISP-1, markers related to cartilage metabolism, showed differential alterations. These findings are consistent with the possibility that reduced MaR1 is associated with altered inflammation-resolution biology in arthritic disorders and support MaR1 as a candidate biomarker associated with these pathways.

**Table 2:** Serum MaR1, COMP, and WISP-1 Levels and Intergroup Comparisons in Rheumatoid Arthritis, Osteoarthritis, and Control Groups.

Biomarker	Control (Mean ± SD)	OA (Mean ± SD)	RA (Mean ± SD)	F (2,147)	*p*-value	η^2^
MaR1 (ng/L)	78.5 ± 7.5	42.2 ± 3.2^a^	44.1 ± 2.2^a^	19.654	< 0.001	0.211
COMP (ng/mL)	58.9 ± 0.3	57.9 ± 0.3	56.7 ± 0.3^a, b^	9.601	< 0.001	0.116
WISP-1 (pg/mL)	1959.4 ± 169.5	1461.7 ± 101.4^a^	1392.1 ± 145.3^a^	4.802	0.013	0.061

Values are expressed as mean ± standard deviation. One-way ANOVA followed by Bonferroni-adjusted post-hoc comparisons.

^a^*p* < 0.05 vs. Control; ^b^*p* < 0.05 vs. OA. Effect sizes are reported as eta-squared (η^2^). Mean differences are shown relative to controls with corresponding 95% confidence intervals (CI).

**Fig. 2:**
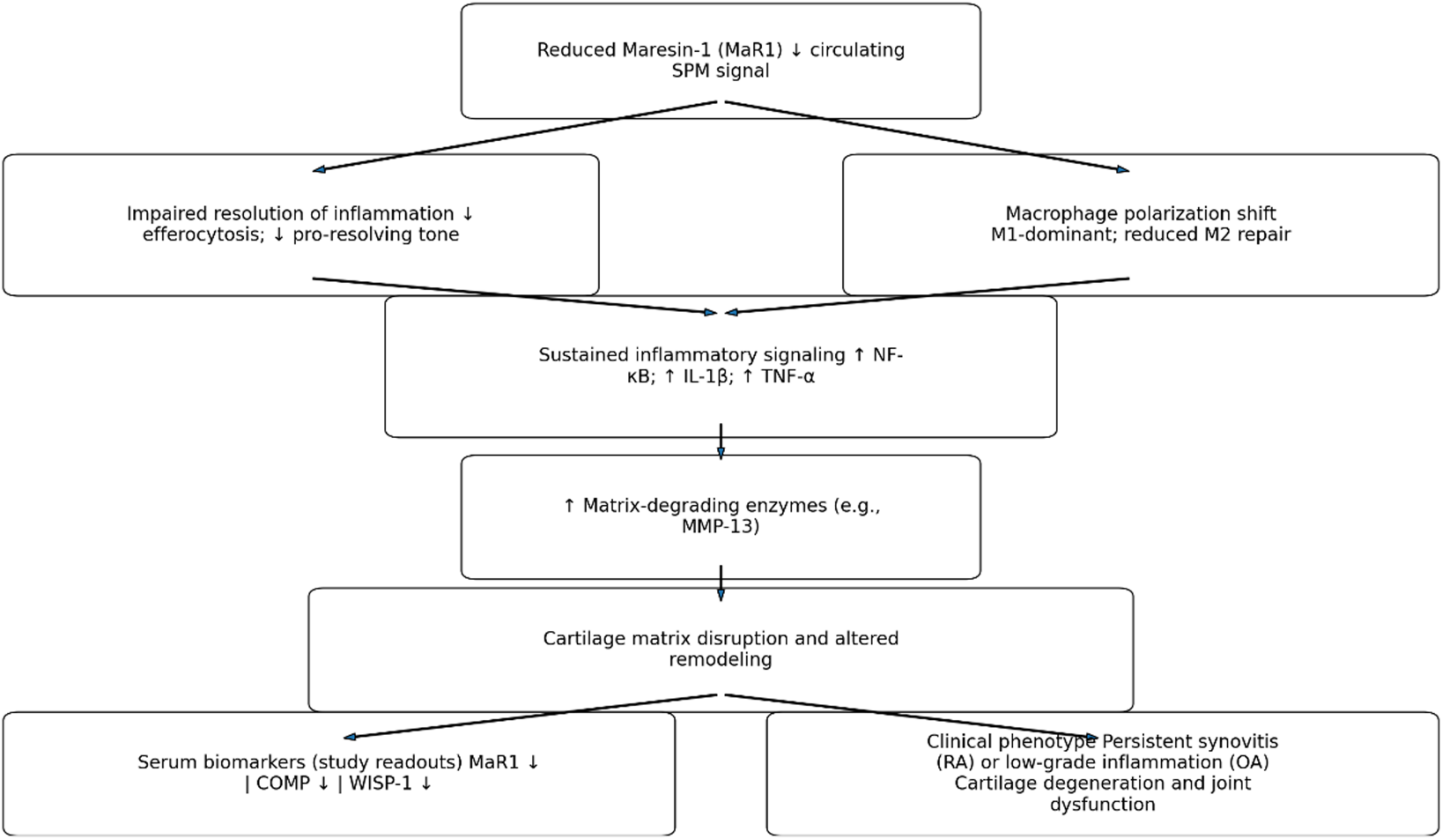
Conceptual model linking reduced Maresin-1 (MaR1) levels to impaired inflammation resolution and altered cartilage remodeling in rheumatoid arthritis and osteoarthritis. Reduced MaR1 may reflect impaired specialized pro-resolving mediator activity, contributing to sustained inflammatory signaling (e.g., NF-κB activation and increased IL-1β and TNF-α), macrophage polarization imbalance, and enhanced matrix-degrading enzyme activity (e.g., MMP-13). These processes may disrupt cartilage matrix homeostasis, consistent with the observed alterations in serum COMP and WISP-1 levels.

The proposed mechanistic framework integrating reduced MaR1 levels with persistent inflammatory signaling and altered cartilage-related biomarkers is summarized in Fig. 2.

**Fig. 3:**
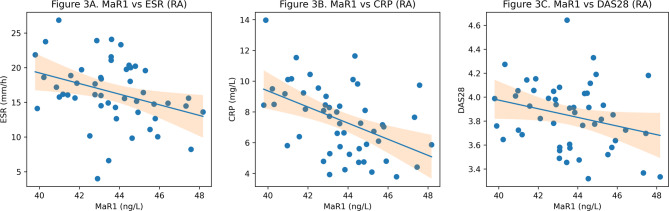
Correlation analyses between serum MaR1 levels and clinical parameters in the rheumatoid arthritis (RA) cohort. (**A**) Relationship between MaR1 and erythrocyte sedimentation rate (ESR). (**B**) Association between MaR1 and C-reactive protein (CRP). (**C**) Correlation between MaR1 and Disease Activity Score 28 (DAS28). Scatter plots display individual patient data points. Solid lines represent linear regression fits, and shaded areas indicate 95% confidence intervals. Pearson correlation coefficients (r) and corresponding p-values are reported in the Results section.

Correlation analyses conducted separately in the RA and OA groups are summarized in Table 3. In the RA cohort, serum MaR1 levels demonstrated significant inverse correlations with ESR (*r* = − 0.42, *p* < 0.01), CRP (*r* = − 0.47, *p* < 0.01), and DAS28 scores (*r* = − 0.38, *p* < 0.01). As shown in Fig. 3, serum MaR1 levels demonstrated significant inverse correlations with ESR, CRP, and DAS28 in the RA cohort. In the OA cohort, MaR1 levels were modestly inversely correlated with ESR (*r* = − 0.29, *p* < 0.05) and CRP (*r* = − 0.28, *p* < 0.05).

COMP levels showed a weak inverse correlation with disease duration in RA patients (*r* = − 0.27, *p* < 0.05), whereas WISP-1 levels demonstrated a mild inverse association with CRP in RA (*r* = -0.26, *p* < 0.05). No significant correlations were observed for these parameters in the OA cohort.

Inter-biomarker correlation analyses revealed that MaR1 levels were weakly inversely correlated with WISP-1 in the RA cohort (*r* = -0.21, *p* < 0.05), whereas no significant association was observed between MaR1 and COMP. No strong correlations were detected between COMP and WISP-1 levels in either disease group. Inter-biomarker correlation analyses did not demonstrate strong direct associations between MaR1, COMP, and WISP-1, suggesting that these mediators may reflect related but partially independent aspects of inflammatory and remodeling biology.

**Table 3:** Correlation coefficients between serum MaR1, COMP, and WISP-1 levels and clinical parameters in rheumatoid arthritis (RA) and osteoarthritis (OA) patients.

Biomarker	Clinical parameter	RA (*r*)	RA (*p*)	OA (*r*)	OA (*p*)
MaR1	ESR	–0.42	< 0.01	–0.29	< 0.05
MaR1	CRP	–0.47	< 0.01	–0.28	< 0.05
MaR1	DAS28	–0.38	< 0.01	—	—
COMP	Disease duration	–0.27	< 0.05	–0.21	> 0.05
WISP-1	CRP	–0.26	< 0.05	–0.18	> 0.05

## Discussion

This study provides insight into the association between MaR1 and serum cartilage remodeling biomarkers in RA and OA. Both diseases are driven by complex inflammatory cascades that culminate in cartilage destruction, joint dysfunction, and chronic pain. Traditional therapeutic strategies primarily aim to suppress inflammation; however, recent discoveries in lipid-mediator biology emphasize resolution as an active, orchestrated process essential for restoring tissue homeostasis rather than merely terminating inflammation^5^. Within this framework, MaR1 has attracted considerable attention for its dual anti-inflammatory and pro-resolving functions, which enable it to regulate immune-cell activity, limit tissue injury, and promote repair^7^.

The present findings demonstrate reduced circulating MaR1 levels in both RA and OA, accompanied by alterations in COMP and WISP-1. These associations may reflect perturbations in inflammation-resolution pathways in chronic joint disease. However, given the study’s cross-sectional design, such interpretations remain speculative. Further mechanistic and longitudinal investigations are required to clarify the biological significance of these observations.

The demographic and clinical characteristics summarized in Table 1 provide the essential background for interpreting biochemical findings. Both OA and RA groups exhibited higher mean ages (61.5 ± 9.5 and 59.0 ± 9.5 years, respectively) than controls (51.2 ± 9.5 years), consistent with the age-related prevalence of degenerative and autoimmune arthropathies. The predominance of females especially in the RA cohort (74%) aligns with the recognized sex bias in inflammatory arthritis, driven by hormonal and genetic factors. Aging is known to influence specialized pro-resolving mediator biosynthesis, systemic inflammatory tone, and extracellular matrix turnover. Although age-adjusted analyses confirmed the persistence of intergroup differences, age-related immunometabolic alterations may partially modulate circulating biomarker levels and should therefore be considered when interpreting systemic measurements.

The higher BMI values in OA (27.5 ± 3.1 kg/m^2^) and RA (26.9 ± 2.8 kg/m^2^) versus controls (24.8 ± 2.3 kg/m^2^) emphasize the combined metabolic and mechanical contributions to joint pathology. Elevated ESR, CRP, and RF values in RA confirm active systemic inflammation, while moderate ESR/CRP increases in OA support its inflammatory component. These findings frame the subsequent interpretation of altered pro-resolving and cartilage-related biomarkers (Table 2).

Importantly, multivariable analyses confirmed that the observed reductions in MaR1 levels were independently associated with disease status rather than sex or BMI. Although sex-related immunological differences and obesity-associated metabolic inflammation are known to influence inflammatory mediator profiles, these factors did not independently account for the biomarker alterations observed in this cohort. This finding strengthens the interpretation that impaired resolution biology is intrinsically linked to arthritic pathology rather than secondary demographic influences.

The most salient observation of this study is the marked reduction in circulating MaR1 concentrations in both RA (44.1 ± 2.2 ng/L) and OA (42.2 ± 3.2 ng/L) compared with controls (78.5 ± 7.5 ng/L; *p* < 0.001), representing an approximate 44% decrease. This deficiency indicates an impairment in endogenous pro-resolving capacity, consistent with reports that SPMs are dysregulated in chronic inflammation^17^. MaR1, a docosahexaenoic acid (DHA)-derived lipid mediator, orchestrates the active return to homeostasis rather than the passive cessation of inflammation^7,23^. Impaired biosynthesis due to reduced activity of SPM-synthetic enzymes or altered omega-3/omega-6 metabolism may limit MaR1 production. Additionally, the hyperinflammatory milieu of RA and OA may accelerate MaR1 consumption by macrophages and neutrophils attempting to counteract persistent inflammation^18^.

Beyond its anti-inflammatory role, MaR1 modulates the immune equilibrium between regulatory T cells (Tregs) and T helper 17 cells (Th17), an axis central to RA pathogenesis^19,20^. By restoring the Treg/Th17 balance through microRNA-21-mediated pathways, MaR1 limits autoreactive activation and promotes immune tolerance. It also suppresses pro-inflammatory cytokines such as IL-1β and TNF-α by inhibiting the NF-κB cascade^21^. Moreover, MaR1 reprograms macrophages from pro-inflammatory M1 toward reparative M2 phenotypes, facilitating apoptotic-cell clearance and synovial recovery^22^. Preclinical studies have reported that MaR1 administration attenuates arthritis severity in experimental models, partly by modulating MMP-13 expression and enhancing type II collagen synthesis^8^. However, the present cross-sectional study does not evaluate functional or interventional effects. The observed reduction in circulating MaR1 levels may therefore reflect an association with inflammatory activity and cartilage remodeling disturbances rather than a demonstrated causal mechanism. Figure 2 provides a conceptual schematic integrating the present biomarker findings with established inflammatory and pro-resolving signaling pathways. This model is intended as a hypothesis-generating framework and should not be interpreted as evidence of direct mechanistic interaction.

The inverse correlations observed between MaR1 levels and inflammatory markers, particularly in the RA cohort, further support the hypothesis that impaired resolution biology is linked to ongoing inflammatory burden. The stronger associations observed in RA compared with OA may reflect the more pronounced systemic inflammatory component of autoimmune arthritis. Although causality cannot be established in this cross-sectional design, the correlation patterns reinforce the potential utility of MaR1 as a biomarker reflecting inflammatory intensity and disease activity. COMP, a pentameric glycoprotein in the cartilage extracellular matrix, serves as a sensitive indicator of cartilage turnover and destruction^10^. Although elevated COMP levels are typically linked to disease progression, the present study revealed significantly lower serum COMP in RA (56.7 ± 0.3 ng/mL) compared with both OA (57.9 ± 0.3 ng/mL) and controls (58.9 ± 0.3 ng/mL; *p* < 0.001). Although the absolute numerical differences were modest, their statistical consistency suggests mechanistic relevance. This decline may reflect long-term disease or pharmacologic suppression of matrix turnover by DMARDs and biologic agents^24^. In late-stage RA, extensive cartilage loss may also reduce the substrate available for COMP release. The concurrent decrease in both MaR1 and COMP levels suggests a functional linkage between defective resolution signaling and altered cartilage metabolism. SPMs can inhibit proteolytic enzymes and inflammatory mediators responsible for matrix degradation, implying that reduced MaR1 bioavailability could indirectly accelerate cartilage damage by deregulating MMP and cytokine pathways^8^.

Notably, the absence of a statistically significant difference in serum COMP levels between the OA and control groups, together with the reduction observed in the RA cohort, warrants careful interpretation. Several studies have reported higher circulating COMP levels in OA, particularly in early or actively progressive disease, and have linked serum COMP to structural damage and/or radiographic progression^10,11^. In contrast, circulating COMP levels may vary according to disease stage, structural severity, and matrix turnover dynamics. During phases of active cartilage degradation, increased COMP release may be observed, whereas in more advanced stages characterized by substantial cartilage loss and reduced substrate availability, circulating levels may attenuate.

The absence of imaging-based structural stratification in the present study limits direct comparison with progression-focused investigations and may partly account for the observed discrepancies. These differences likely reflect variability in cohort characteristics, disease-stage distribution, and inflammatory phenotype, underscoring the heterogeneity of biomarker expression across distinct clinical contexts of joint disease.

Importantly, even in the absence of statistical significance, the observed directional trend may reflect underlying biological heterogeneity across OA stages. Therefore, interpretation should integrate effect size estimates and biological plausibility rather than relying exclusively on p-values.

In contrast, the lower COMP levels observed in RA patients require cautious interpretation, as elevated COMP concentrations have frequently been associated with active cartilage degradation in inflammatory arthritis. Although chronic disease duration and treatment-related modulation of cartilage turnover may influence circulating COMP levels, detailed radiographic damage assessments and treatment-stratified analyses were not available in the present study. Therefore, the reduced COMP concentrations observed in RA should be considered hypothesis-generating rather than definitive and may reflect variability in structural severity, treatment exposure, or matrix turnover dynamics.

WISP-1, a CCN-family matricellular protein downstream of Wnt/β-catenin signaling, regulates chondrocyte differentiation, extracellular matrix remodeling, and synovial inflammation^13^. While prior studies have reported elevated WISP-1 expression in arthritic tissues, our results show reduced serum WISP-1 levels in both OA (1461.7 ± 101.4 pg/mL) and RA (1392.1 ± 145.3 pg/mL) compared with controls (1959.4 ± 169.5 pg/mL; *p* = 0.013). This discrepancy between local tissue overexpression and systemic reduction may stem from compartmentalization, altered clearance, or timing of sampling relative to disease activity. Treatment exposure might also contribute to systemic WISP-1 differences, although medication-stratified analyses were not available in the present study. The parallel decrease across RA and OA, despite distinct etiologies, suggests that WISP-1 dysregulation represents a shared molecular signature of chronic joint degeneration.

While increased WISP-1 expression has been reported in synovial tissue and cartilage samples from patients with RA and OA, and elevated serum COMP has been associated with disease progression in specific cohorts, our study demonstrated reduced circulating concentrations in patients with established disease. Several previous investigations have documented increased COMP and WISP-1 levels, particularly during early or actively progressive stages^11,14,15^. The apparent discrepancy may reflect differences in disease duration, therapeutic exposure, and the biological compartment analyzed. Many prior studies focused on synovial tissue or fluid measurements, which may not directly parallel systemic circulating concentrations.

In advanced or long-standing disease, progressive cartilage loss and diminished active matrix turnover may alter biomarker release kinetics, potentially contributing to lower circulating COMP levels. Likewise, chronic modulation of inflammatory and Wnt/β-catenin signaling pathways by DMARDs or biologic therapies may attenuate systemic WISP-1 concentrations. Thus, rather than representing a direct contradiction of existing literature, our findings may reflect stage-dependent and compartment-specific remodeling dynamics in chronic arthritic conditions.

A potential interaction between MaR1 and WISP-1-related pathways may be hypothesized based on previous experimental observations. While MaR1 has been reported to modulate inflammatory signaling pathways, including NF-κB, WISP-1 has been experimentally implicated in cartilage degeneration through Wnt/CCN-related mechanisms^25^. However, the present study does not provide direct mechanistic evidence of MaR1–WISP-1 crosstalk. The observed inverse associations are derived from circulating biomarker measurements and therefore do not establish functional or regulatory interaction. Accordingly, any proposed relationship between these mediators should be considered hypothetical and requires validation in experimental or pathway-based models.

The concurrent alterations in MaR1, COMP, and WISP-1 levels further suggest an interconnection between defective inflammation resolution and impaired matrix remodeling. MaR1 can suppress MMP-13 through activation of the PI3K/AKT pathway and inhibition of NF-κB signaling in synoviocytes, directly linking resolution deficiency to cartilage degradation^8^. Therapeutically, this MaR1 deficiency implies that enhancing pro-resolving capacity could complement existing treatments. Unlike conventional anti-inflammatory agents, MaR1-based strategies promote active resolution without impairing host defense^26^. Incorporating SPM assessments into clinical monitoring may refine personalized treatment planning. Moreover, baseline MaR1 profiles have been shown to predict DMARD responsiveness in early RA^17^. The integration of MaR1 with traditional biomarkers and imaging could thus improve prognostic precision. The interrelationships between MaR1, COMP, and WISP-1 indicate a coordinated dysregulation of inflammation resolution and cartilage remodeling pathways, reinforcing their potential as complementary biomarkers in arthritis pathophysiology.

This study has several limitations. First, its cross-sectional design precludes causal inference regarding biomarker alterations and disease progression. Although age was statistically adjusted for, the younger control group may still introduce residual confounding. In addition, the potential influence of ongoing pharmacotherapy on biomarker dynamics was not systematically evaluated and may partly account for the observed variability. Another important consideration is the potential influence of ongoing pharmacologic therapy on circulating biomarker levels. Patients with RA were under chronic DMARD and/or biologic treatment, which may modulate inflammatory and cartilage-related pathways. Although sampling was performed under clinically stable conditions, residual treatment effects cannot be entirely excluded. Future studies stratifying patients by medication class and treatment duration will provide further clarity. Another important limitation is the exclusive reliance on serum measurements. Joint pathology in RA and OA primarily occurs within local compartments such as the synovium and synovial fluid. Circulating biomarker levels may be influenced by systemic metabolism, clearance mechanisms, and extra-articular inflammatory activity and therefore may not precisely reflect intra-articular concentrations. Paired analyses of serum and synovial fluid or synovial tissue samples would provide more comprehensive insight into compartment-specific regulation of MaR1, COMP, and WISP-1.

Future research should incorporate longitudinal designs, strictly age-matched cohorts, and simultaneous local and systemic sampling to clarify compartmentalization effects. Interventional studies assessing synthetic MaR1 analogues or specialized pro-resolving mediator supplementation, together with mechanistic in vitro investigations in chondrocytes and synoviocytes, would further elucidate the causal role of resolution pathways in arthritis.

Taken together, the present findings should be interpreted as hypothesis-generating and provide a clinical framework for future resolution-based investigations in inflammatory and degenerative joint diseases.

## Conclusions

This study demonstrates an association between reduced circulating MaR1 levels and arthritic conditions, accompanied by alterations in cartilage-related biomarkers. These findings suggest that dysregulation of inflammation-resolution pathways may be linked to chronic joint pathology and cartilage remodeling dynamics. However, given the study’s cross-sectional design, causal relationships cannot be established. Further longitudinal and mechanistic investigations are required to determine whether modulation of MaR1 pathways has translational or clinical relevance.

## Supplementary Information

Below is the link to the electronic supplementary material.

Supplementary Material 1

## Data Availability

The datasets generated and analyzed during the current study are available from the corresponding author upon reasonable request.
