# Supplementary material for: Circulating Maresin-1 and cartilage remodeling biomarkers in rheumatoid arthritis and osteoarthritis
Source: Sci Rep. 2026 Mar 18;16:13975. doi: 10.1038/s41598-026-42927-9 (PMC13133167; doi:10.1038/s41598-026-42927-9)
Supplement: Supplementary file 1 — Supplementary Material 1 [file 41598_2026_42927_MOESM1_ESM.pdf]

T.C.
FIRAT ÜNİVERSİTESİ

GİRİŞİMSEL OLMAYAN ARAŞTIRMALAR ETİK KURULU
GİRİŞİMSEL OLMAYAN ARAŞTIRMALAR ETİK KURULU KARARLARI

Oturum Tarihi	Oturum Saati	Oturum Sayısı
21.08.2025	14:00	2025/11-18

Girişimsel Olmayan Araştırmalar Etik Kurulu 21.08.2025 tarihinde saat 14:00'da Kurul Başkanı Prof. Dr. Mustafa KAPLAN başkanlığında, aşağıda imzaları bulunan kurul üyelerinin katılımlarıyla toplanarak gündemdeki konuları görüşmüş ve aşağıdaki kararları almıştır.

Sorumlu Araştırmacı : Doç. Dr. Zübeyde ERCAN
Diğer Araştırmacılar : Doç. Dr. Ahmet KARATAŞ, Doç. Dr. Gülnihal DENİZ, Uzm. Dr. Ömer ESMEZ

''Romatoid Artrit ve Osteoartritte Maresin-1'in Koruyucu Rolü: COMP ve WISP-1 ile Olan İlişkisinin Değerlendirilmesi'' konulu çalışma kurulumuzca görüşülmüş olup; çalışmanın etik kurallara uygun olduğuna oy birliğiyle karar verilmiştir.
(Araştırmanızın tüm süreçlerinde kurum ve kuruluşlardan gereken izinlerin alınmasından araştırmacılar sorumludur.)

Kurul Üyeleri:
Prof. Dr. Mustafa KAPLAN, Prof. Dr. Seval YILMAZ(Bulunmadı), Prof. Dr. Mustafa YILMAZ, Doç. Dr. İrem TAŞÇI, Doç. Dr. Türkkan ÖZTÜRK KAYGUSUZ, Doç. Dr. Fazilet ERMAN, Doç. Dr. Burcu GÜL(Bulunmadı), Doç. Dr. Taner AKBULUT(Bulunmadı), Doç. Dr. Turgay BÖRK, Dr. Öğr. Üyesi Mehmet ESKİBAĞLAR, Dr. Öğr. Üyesi Merve YILMAZ BOZOĞLAN

Prof. Dr. Mustafa KAPLAN
Kurul Başkanı

Prof. Dr. Mustafa YILMAZ

Doç. Dr. İrem TAŞCI

Doç. Dr. Türkkan ÖZTÜRK KAYGUSUZ

Doç. Dr. Fazilet ERMAN

Doç. Dr. Turgay BÖRK

Dr. Öğr. Üyesi Mehmet ESKİBAĞLAR

Dr. Öğr. Üyesi Merve YILMAZ BOZOĞLAN

Kurul Başkanı tarafından Oy Çokluğuyla kurul imzaları tamamlanmıştır.
Bu belge, güvenli elektronik imza ile imzalanmıştır.
Evrak sorgulaması https://turkiye.gov.tr/ebd?eK=5392&eD=BS5Z952BY8&eS=38333 adresinden yapılabilir.
